# The Effects of Caudal Epidural Injection on Optic Nerve Sheath Diameter and Hemodynamic Parameters in Adults with Failed Back Surgery Syndrome: A Prospective Clinical Study

**DOI:** 10.5812/aapm-166101

**Published:** 2025-11-04

**Authors:** Mehrdad Taheri, Sohrab Salimi, Alireza Jaffari, Payman Dadkhah, Alireza Shakeri, Mohammad-Reza Razavizade

**Affiliations:** 1 Department of Anesthesiology, Imam Hossein Hospital, School of Medicine, Shahid Beheshti University of Medical Sciences, Tehran, Iran; 2 Anesthesiology Research Center, Imam Hossein Hospital, School of Medicine, Shahid Beheshti University of Medical Sciences, Tehran, Iran; 3 Department of Anesthesiology, Trauma Research Center, Kashan University of Medical Sciences, Kashan, Iran

**Keywords:** Failed Back Surgery Syndrome, Caudal Epidural Injection, Optic Nerve Sheath Diameter, Intracranial Pressure, Hemodynamic Monitoring, Ultrasonography

## Abstract

**Background:**

Failed back surgery syndrome (FBSS) is a challenging chronic pain condition following spinal surgery, often resistant to conventional therapies. Caudal epidural injection is a mainstay for managing FBSS, yet its effects on intracranial pressure (ICP), particularly in adults with post-surgical anatomical changes, remain poorly understood. Optic nerve sheath diameter (ONSD) measured by ultrasound offers a non-invasive surrogate marker for detecting alterations in ICP.

**Methods:**

This prospective single-center clinical trial enrolled 46 adult FBSS patients scheduled for therapeutic caudal epidural injection at Imam Hussein Hospital, Tehran, Iran. Each participant received a standardized two-stage, 30 mL caudal epidural injection. The ONSD and hemodynamic parameters [systolic, diastolic and mean arterial blood pressure (SBP), (DBP), (MAP), heart rate (HR)] were assessed at baseline, immediately, and then at 10, 20, and 40 minutes post-injection. All measurements were performed by blinded, trained personnel using validated protocols.

**Results:**

Caudal epidural injection produced a significant, transient increase in mean ONSD (baseline: 4.8 ± 0.49 mm; immediate post-injection: 5.1 ± 0.50 mm; P < 0.001), which normalized within the observation period. No patient exhibited symptoms or clinical signs of raised ICP. While serial monitoring indicated statistically significant reductions in SBP, DBP, MAP, and HR at 40 minutes, all values remained within physiologically acceptable ranges. No major procedural complications or adverse neurological outcomes occurred.

**Conclusions:**

Standard-volume caudal epidural injection in adults with FBSS causes a temporary, asymptomatic elevation in ONSD, reflecting a reversible change in ICP. The procedure was well tolerated, with minimal and clinically insignificant hemodynamic effects, supporting its safety and utility in this patient population.

## 1. Background

Failed back surgery syndrome (FBSS) is a persistent, debilitating pain condition that manifests after spinal surgery, representing a significant clinical and socioeconomic challenge worldwide. Despite advances in neurosurgical and orthopedic techniques, the incidence of FBSS has remained substantial, with prevalence estimates ranging from 10% to 40% among individuals undergoing lumbar spine surgery (1, 2). The syndrome is defined by chronic pain localized in the lower back and/or legs, either persisting or recurring after surgical intervention, and is often unresponsive to conventional treatments. Typical management strategies range from pharmacologic therapy and physical rehabilitation to repeated surgical interventions, with an increasing emphasis on interventional pain management procedure (3-6).

Among these interventions, epidural injections — particularly via the caudal route — are frequently utilized for both diagnostic and therapeutic purposes. Caudal epidural injection allows for the administration of medications, including corticosteroids, local anesthetics, hyaluronidase, and hypertonic saline, directly into the epidural space with the aim of reducing inflammation, lysing adhesions, and alleviating neuropathic pain (7).

Despite its widespread clinical adoption, the physiological consequences of caudal epidural injection — especially when large volumes are administered — have not been fully elucidated. Of particular concern is the potential impact on cerebrospinal fluid (CSF) dynamics and intracranial pressure (ICP), areas that are especially relevant in patients with altered spinal anatomy, scarring, or changes in epidural compliance, such as those found in FBSS.

**Figure 1.**
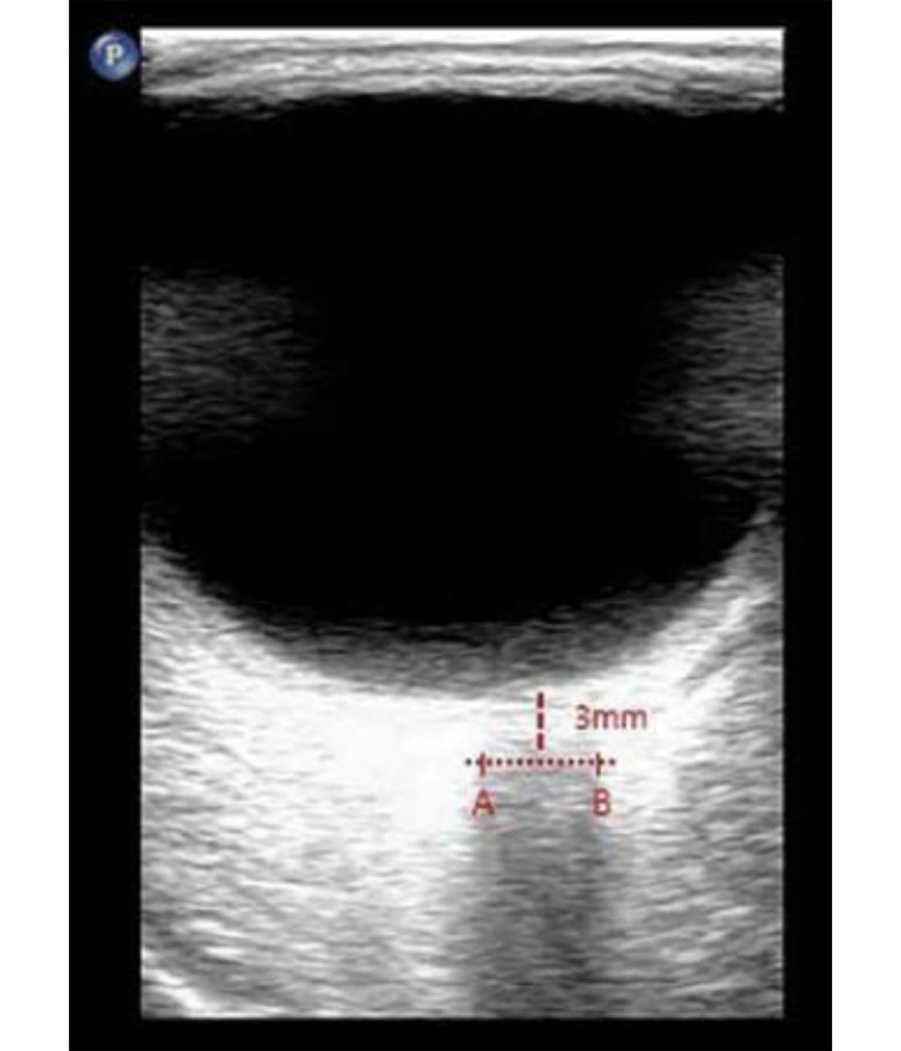
Optic nerve sheath diameter (ONSD) measurement by ultrasonography. The ONSD was measured by 3 mm posterior to the optic nerve head (A and B) in the axial image of the orbit in the plane of the optic nerve.

Direct invasive monitoring of ICP, the gold standard for assessment, is not feasible in routine interventional pain practice. Consequently, there is growing interest in the use of non-invasive surrogate markers to assess changes in ICP in real time. Among these, ultrasonographic measurement of the optic nerve sheath diameter (ONSD) has emerged as a validated and reliable approach (8-10). The optic nerve sheath, which is contiguous with the subarachnoid space, serves as a sensitive indicator; increases in ICP are rapidly transmitted to this compartment, resulting in measurable sheath expansion, particularly 3 mm behind the globe where distensibility is greatest (Figure 1). 

Recent evidence, primarily derived from pediatric anesthesia and neurosurgical populations, suggests that neuroaxial blocks, including caudal and thoracic epidural injections, can transiently increase ONSD, with changes paralleling acute ICP elevations (8, 9). However, there is a paucity of robust data describing these effects in adults, and even fewer studies have specifically addressed the FBSS population — a group with unique anatomical and physiological characteristics resulting from previous spinal surgery and chronic pain pathology.

We undertook a prospective clinical investigation to systematically evaluate the effect of caudal epidural injection volume on ONSD as a non-invasive ICP marker in adult patients with FBSS. Secondary endpoints included serial assessment of systolic and diastolic blood pressure (SBP, DBP), mean arterial pressure (MAP), and heart rate (HR) to provide a comprehensive evaluation of physiological responses post-intervention.

## 2. Methods

### 2.1. Study Design and Setting

This prospective, single-center clinical trial was conducted at the Pain Clinic of Imam Hussein Hospital, affiliated with Shahid Beheshti University of Medical Sciences, Tehran, Iran, between January 2023 and January 2024. The study protocol was reviewed and approved by the institutional review board (IR.SBMU.RETECH.REC.1403.077) and was registered with the Iranian Registry of Clinical Trials (IRCT20240609062049N1). All participants provided written informed consent prior to enrollment in accordance with the Declaration of Helsinki.

### 2.2. Participants

#### 2.2.1. Inclusion and Exclusion Criteria

Eligible participants were adults aged 18 - 80 years who had been diagnosed with FBSS, defined as the presence of chronic low back and/or leg pain persisting for more than six months following lumbar spinal surgery. All participants were scheduled to undergo a therapeutic caudal epidural injection at our institution. Exclusion criteria comprised of (A) History of coagulopathy or ongoing anticoagulant therapy; (B) known or suspected intracranial pathology; (C) active systemic or local infection at the injection site; (D) pre-existing optic nerve or ocular disease, (E) clinical diagnosis of cauda equina syndrome; (F) cerebrovascular disease; (G) pregnancy or breastfeeding.

#### 2.2.2. Sample Size Calculation

Sample size estimation was based on prior literature reporting a mean increase of 0.37 mm in ONSD following neuroaxial interventions, with a pooled standard deviation (SD) of 0.37 mm (11). To achieve a statistical power of 90% (β = 0.10) and a significance level of 0.05 (α = 0.05), a minimum of 46 participants was deemed necessary, considering potential attrition.

### 2.3. Data Collection and Baseline Assessment

Upon enrollment, demographic and clinical data — including age, sex, height, weight, and Body Mass Index (BMI) — were recorded. Baseline measurements of ONSD, SBP, DBP, MAP and HR were obtained with patients in the supine position and under standardized conditions.

#### 2.3.1. Procedure

Participants were positioned prone with a pillow placed under the pelvis to facilitate access to the sacral hiatus. Intravenous access was secured, and routine monitoring — including noninvasive blood pressure, pulse oximetry, and continuous electrocardiography — was maintained throughout the procedure.

Under strict aseptic precautions and ultrasound guidance (high-frequency linear transducer, 7 - 13 MHz), a 22-gauge Quincke spinal needle was advanced via the caudal approach into the epidural space. All injections were performed by a single experienced pain specialist to minimize inter-operator variability. Each patient received a total volume of 30 mL caudal epidural injectate, administered in two stages:

- The first injectate included 10 mL of normal saline containing 1,500 units of hyaluronidase and 10 mL of 0.2% ropivacaine with 40 mg triamcinolone acetate.

- After an interval of 20 minutes, a further 10 mL of 5% hypertonic saline was injected.

Injection rates were carefully controlled to avoid excessive pressure; the two-stage protocol was designed to minimize abrupt shifts in epidural and ICP. Throughout the procedure and the subsequent monitoring period, patients were observed for adverse events including headache, visual symptoms, nausea, dizziness, or significant fluctuations in vital signs.

#### 2.3.2. Optic Nerve Sheath Diameter Measurement

The ONSD was measured in supine position, at baseline (pre-injection), immediately after the initial injection, and subsequently at 10, 20, and 40 minutes post-injection. A single, trained, and blinded sonographer performed all measurements using a high-frequency (7 - 13 MHz) linear array ultrasound probe. Measurement technique was as follows:

- The patient’s eyes remained closed, and ample ultrasound gel was applied to avoid pressure on the globe.

- Bilateral ONSD was assessed in a transverse axial plane, 3 mm posterior to the optic disc, which is considered the most sensitive point for detecting changes in ICP (8-10).

- For each session, two measurements were taken per eye and averaged, with the final value expressed as the mean of both eyes at each time point (8-10).

This protocol is consistent with established international guidelines and has demonstrated high reproducibility and accuracy.

#### 2.3.3. Hemodynamic Monitoring

The SBP, DBP, MAP and HR were recorded at the same time-points as ONSD measurements: At baseline, immediately post-injection, and at 10, 20, and 40 minutes following the procedure. Any significant deviation in vital signs or the occurrence of complications were documented and managed according to institutional protocols.

### 2.4. Statistical Analysis

All data were managed and analyzed using IBM SPSS Statistics for Windows (Version 27.0.1; IBM Corp., Armonk, NY, USA). Continuous variables are reported as mean ± SD, while categorical variables are summarized as counts and percentages. For variables with repeated measurements (ONSD, SBP, DBP, MAP, HR), repeated measures analysis of variance (ANOVA) was employed to assess changes over time. Post hoc pairwise comparisons were conducted with Bonferroni correction where appropriate. A P < 0.05 was considered indicative of statistical significance. The presence of any missing data and handling procedures were explicitly documented.

## 3. Results

A total of 46 adult patients with FBSS were enrolled and completed the study protocol. The demographic and baseline clinical features of the cohort are summarized in Table 1. The mean age of participants was 59.1 ± 8.5 years (range: 18 - 80 years), and the study population consisted of 16 males (34.8%) and 30 females (65.2%). The average BMI was 28.4 ± 2.4 kg/m^2^. No participant was lost to follow-up or withdrew from the protocol, and all were included in the final analysis.

**Table 1.** Demographic Characteristics of Patients with Failed Back Surgery Syndrome Undergoing Caudal Epidural Injection ^a^

Variables	Status
**Gender**	
Male	16 (34.8)
Female	30 (65.2)
**Age (y)**	59.1 ± 8.5
**Weight (kg)**	78.6 ± 8.9
**Height (m)**	1.66 ± 9.9
**BMI (kg/m** ^**2**^ **)**	28.4 ± 2.4

^a^ Values are expressed as No (%) or mean ± SD.

**Figure 2.**
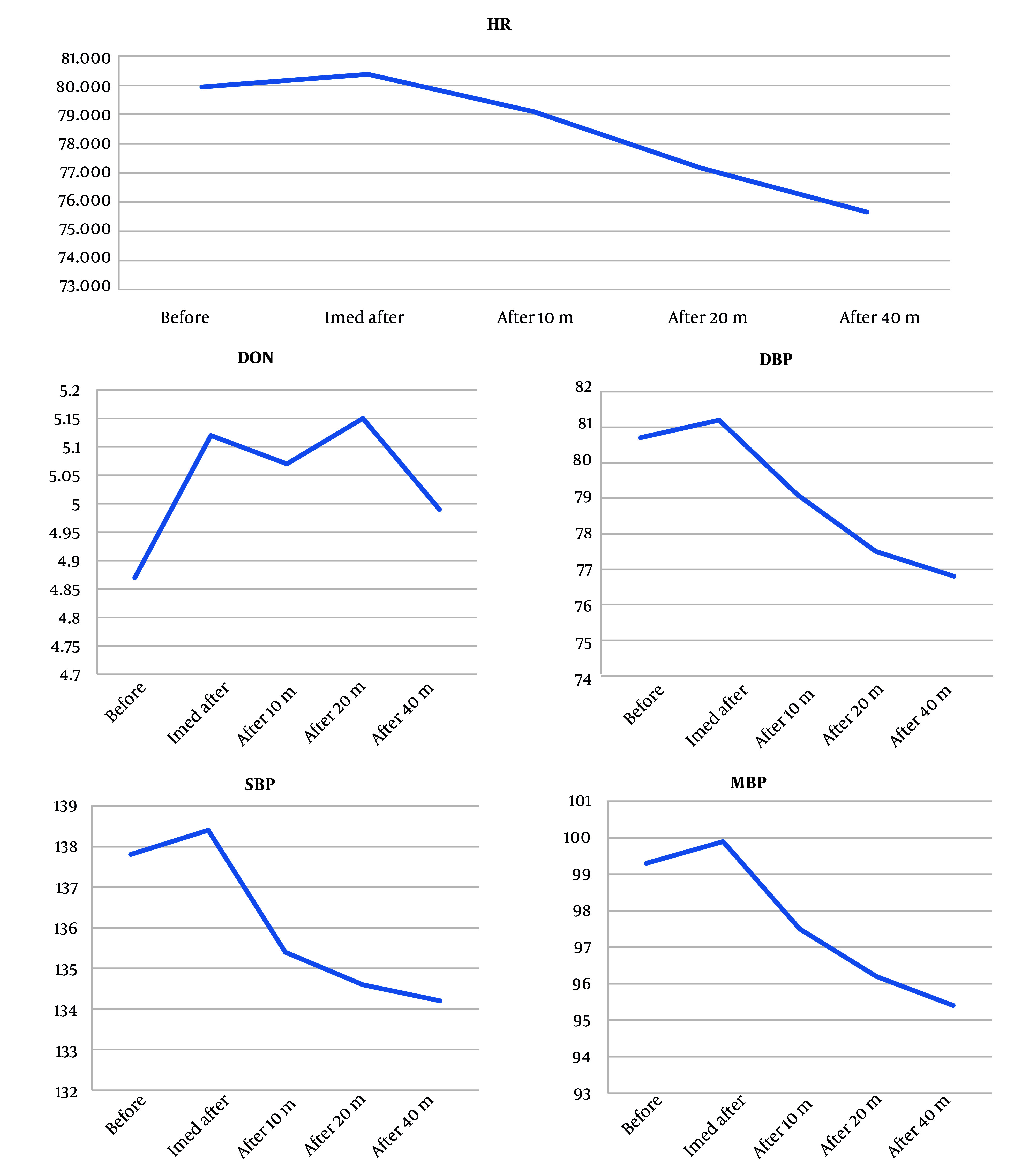
Variation in optic nerve sheath diameter (ONSD), systolic, diastolic and mean arterial blood pressures (SBP), (DBP), and (MAP) and heart rate (HR), throughout the study period

Baseline mean ONSD recorded in the supine position was 4.8 ± 0.49 mm. Following caudal epidural injection of the study solution, mean ONSD increased to 5.1 ± 0.50 mm immediately post-injection, representing a significant change from baseline (P < 0.001). Serial assessments demonstrated that this increase in ONSD was sustained at 10 minutes (mean: 5.1 ± 0.50 mm, P < 0.001), 20 minutes (mean: 5.02 ± 0.47 mm, P < 0.001), and 40 minutes (mean: 4.97 ± 0.47 mm, P < 0.001) after injection (Table 2 and Figure 2). 

**Table 2.** Optic Nerve Sheath Diameter and Hemodynamic Parameters at Different Time Points ^a, b^

Variable (Unit)	T0	TI	T10	T20	T40	P-Value2	P-Value1 (vs T0)
**ONSD (mm)**	4.8 ± 0.49	5.1 ± 0.50	5.1 ± 0.50	5.02 ± 0.47	4.97 ± 0.47	0.015	< 0.001
**SBP (mmHg)**	137.8 ± 16.7	138.4 ± 14.8	135.4 ± 14.6	134.6 ± 14.7	134.2 ± 14.5	< 0.001	0.584
**DBP (mmHg)**	80.7 ± 10.7	81.2 ± 10.2	79.1 ± 10.1	77.5 ± 9.8	76.8 ± 9.3	< 0.001	0.29
**MAP (mmHg)**	99.3 ± 11.9	99.9 ± 10.9	97.5 ± 10.8	96.2 ± 10.7	95.4 ± 10.2	< 0.001	0.291
**HR (bpm)**	79.9 ± 11.1	80.4 ± 11.1	79.1 ± 10.8	77.2 ± 10.4	75.7 ± 9.99	< 0.001	0.549

Abbreviations: ONSD, optic nerve sheath diameter; SBP, systolic blood pressure; DBP, diastolic blood pressure; MAP, mean arterial blood pressure; HR, heart rate.

^a^ Values are expressed as mean ± SD.

^b^ T0, before caudal epidural injection; TI, immediately after caudal epidural injection; T10, 10 minutes after caudal epidural injection; T20, 20 minutes after caudal epidural injection; T40, 40 minutes after caudal epidural injection; P-value1, comparison of each time point versus T0 (baseline); P-value2, P-value for repeated measures ANOVA across all time points.

Statistical analysis using repeated measures ANOVA confirmed a significant time-dependent effect on ONSD values (P = 0.015). Although all post-injection time points showed elevations compared to baseline, ONSD values remained within the established physiological safety margins. No outlier cases with substantial ONSD elevation were observed. Importantly, none of the patients experienced clinical signs or symptoms indicative of elevated ICP, such as headache, altered visual acuity, nausea, vomiting, or changes in mental status during the monitoring period. Assessment for adverse neurological sequelae was systematically negative in all cases. The effects of caudal epidural injection on hemodynamic parameters were closely monitored and are detailed in Table 2. The mean baseline SBP was 137.8 ± 16.7 mmHg, DBP was 80.7 ± 10.7 mmHg, and MAP was 99.3 ± 11.9 mmHg. The HR averaged 79.9 ± 11.1 beats per minute at baseline.

Immediately after injection, no significant changes in SBP, DBP, or MAP were detected (all P > 0.05). However, at the 40-minute post-injection assessment, statistically significant reductions were observed for SBP (134.2 ± 14.5 mmHg, P < 0.001), DBP (76.8 ± 9.3 mmHg, P < 0.001), and MAP (95.4 ± 10.2 mmHg, P < 0.001) compared to baseline. Heart rate also demonstrated a meaningful decrease by 40 minutes post-procedure (71.75 ± 9.99 bpm, P < 0.001).

Despite these statistically significant trends, all hemodynamic parameters remained within clinically acceptable ranges for adult patients throughout the observation period. No episodes of hypotension (SBP < 90 mmHg), bradycardia (HR < 50 bpm), or arrhythmia were observed, and no patient required pharmacological or non-pharmacological intervention for cardiovascular instability.

No major adverse events, including infectious complications, allergic reactions, or neurological deterioration, occurred in any participant. There were no cases of post-dural puncture headache, visual disturbances, or deterioration in consciousness. All patients tolerated the procedure and subsequent monitoring without difficulty.

This trial demonstrated that administration of a standard-volume caudal epidural injection (30 mL) in FBSS patients produced a rapid, transient, and statistically significant increase in ONSD, a validated non-invasive surrogate for ICP, without provoking neurological symptoms or clinical evidence of raised ICP. While hemodynamic parameters were observed to decrease slightly by 40 minutes post-injection, all remained within safe clinical limits and none required therapeutic intervention.

## 4. Discussion

This prospective clinical trial investigated the effects of standardized-volume caudal epidural injection on ONSD, a validated non-invasive marker for ICP, in patients with FBSS. The main finding was a significant, yet transient and asymptomatic, increase in ONSD after the injection, while hemodynamic parameters demonstrated slight but clinically innocuous decreases within the 40-minute post-injection window. These results provide important new evidence regarding the safety and physiological effects of caudal epidural interventions in this specific patient group.

### 4.1. Mechanistic Interpretation and Pathophysiological Relevance

Epidural injection of large fluid volumes can generate a transient rise in epidural and CSF pressures, attributable to the anatomical continuity between the epidural and subarachnoid spaces through the dural sheath. The subsequent cranial transmission of this pressure, as theorized in both physiological models and experimental observations (10-12), can result in a temporary elevation of ICP. The ONSD measurement — especially at 3 mm behind the globe, the area most physiologically responsive to ICP shifts — provides a sensitive and reproducible means of tracking these changes (11). Our findings, demonstrating an increase in ONSD after caudal injection, are consistent with this pathophysiological mechanism and concordant with changes reported in pediatric and non-FBSS adult cohorts following neuroaxial blockade or similarly voluminous epidural interventions (8, 9, 13).

The robustness of the observed ONSD changes in our study reinforces the concept that, while caudal epidural injections in adults can result in measurable alterations in surrogate ICP markers, these effects are transient and self-limited when standard protocols and patient selection criteria are respected. The prompt return of ONSD to baseline in all cases, along with the lack of clinical symptoms suggestive of intracranial hypertension, provides assurance regarding the neurological safety of the procedure. This may be attributed to several mitigating factors:

- Slow/staged injection protocol: The administration of solutions in staggered intervals likely attenuated the peak epidural and CSF pressure elevations.

- Postsurgical epidural changes: Patients with FBSS often display adhesions or altered epidural compliance, which may buffer cranial pressure transmission (14, 15).

- Compensatory CSF mechanisms: Rapid redistribution and reabsorption of CSF, including compensatory drainage via arachnoid villi, contribute to the normalization of transient ICP elevations.

Our findings are also supported by recent MRI-based and sonographic studies in perioperative settings, as well as trials using direct manometry in critical care and anesthesia populations, which demonstrate high levels of agreement between ONSD and invasive ICP readings (11, 12).

The clinical safety observed in our cohort, with no new onset of headache, visual changes, dizziness, or altered consciousness, underlines that in adults without overt risk for raised ICP, caudal epidural injection — even with relatively high volumes — is unlikely to provoke neurologically significant sequelae. Importantly, all measured ONSDs remained below thresholds considered dangerous for acute neurological deterioration (< 5.8 - 6.0 mm in adults), and no delayed symptoms were documented throughout the short-term observation period. These findings are of special clinical relevance, considering the ongoing expansion of interventional pain management techniques for FBSS and the frequent need for high-volume and multi-agent caudal approaches.

In terms of hemodynamic impact, our results indicate that caudal injection is associated with slight but statistically significant falls in SBP, DBP, and MAP, as well as HR, by 40 minutes post-injection. Nonetheless, all measured values stayed within clinically acceptable ranges, and no cases of hypotension, bradycardia, or arrhythmia were encountered, corroborating the safety of the procedure from a cardiovascular perspective. Our observations echo prior reports in other populations undergoing neuroaxial anesthesia, further highlighting the minimal risk profile of this intervention when proper monitoring is observed (10, 12).

While several works have explored ONSD changes following neuroaxial blockade in pediatric cohorts (8, 9, 13), reports in adult populations — and specifically among those with altered spinal anatomy owing to prior surgery — are limited. The current study adds to the sparse literature in this area, confirming that the physiological perturbations observed after caudal injection are quantitatively and temporally similar to those reported in children and non-FBSS adults, and extending these findings to offer reassurance for pain practitioners treating post-surgical spinal patients.

### 4.2. Strengths and Limitations

A central strength of this research lies in its prospective design, standardized procedural and measurement technique, and rigorous, blinded assessment protocols. The use of a single experienced pain specialist and a single trained, blinded sonographer adds to the methodological strength by reducing operator-dependent error. Immediate and serial monitoring of both neurologic and hemodynamic endpoints allows for high-fidelity trend analysis.

However, several limitations must be considered:

- Lack of direct ICP measurement: While ONSD is a well-validated surrogate, invasive ICP monitoring was not performed.

- Single-center, homogeneous cohort: The study was conducted at a single institution on a relatively uniform patient group, potentially affecting generalizability.

- Short monitoring window: The protocol only assessed changes within 40 minutes; longer-term effects were not captured.

- Operator dependence: Despite blinding and protocol standardization, ONSD sonography requires high operator expertise for reproducibility.

Future studies should involve larger, multi-center cohorts, longer observation periods, and might include high-risk patients or those with baseline intracranial pathology to verify the procedural safety across broader populations.

### 4.3. Conclusions

In conclusion, our findings establish that standard-volume caudal epidural injection in FBSS patients is associated with a temporary, clinically silent elevation in ONSD, reflecting a reversible change in ICP without evidence of adverse neurologic or hemodynamic outcomes. These results reinforce the procedural safety and clinical utility of caudal epidural injection in this challenging patient group.

## Data Availability

The datasets supporting the finding of this study are available from the corresponding author upon reasonable request.

## Clinical Trial Registration Code:

IRCT20240609062049N1.
